# Peer-driven quality improvement among health workers and traditional birth attendants in Sierra Leone: linkages between providers’ organizational skills and relationships

**DOI:** 10.1186/1472-6963-15-S1-S4

**Published:** 2015-06-08

**Authors:** Ariel Higgins-Steele, Kathryn Waller, Jean Christophe Fotso, Linda Vesel

**Affiliations:** 1 UNICEF Afghanistan, United Nations Office Complex in Afghanistan, Jalalabad Road, Kabul, Afghanistan; 2 Concern Worldwide US, 355 Lexington Avenue, New York, NY 10017, USA; 3 UNICEF, 3 United Nations Plaza, New York, NY 10017, USA

**Keywords:** Health workforce, quality improvement, peer groups, traditional birth attendants (TBAs), Sierra Leone

## Abstract

**Background:**

Sierra Leone has among the poorest maternal and child health indicators in the world and investments in public health have been predominately to increase demand for services, with fewer initiatives targeting supply side factors that influence health workers’ work environment. This paper uses data from the Quality Circles project in a rural district of Sierra Leone to achieve three objectives. First, we examine the effect of the intervention on organizational skills and relationships among coworkers as well as between health workers and traditional birth attendants. Second, we examine whether changes in organizational skills are associated with changes in relationships among and between formal and informal health providers and between health providers and clients. Third, we aim to further understand these changes through the perspectives of health workers and traditional birth attendants.

**Methods:**

The Quality Circles project was implemented in Kailahun District in the Eastern province of Sierra Leone from August 2011 to June 2013, with adjacent Tonkolili District serving as the control site. Using a mixed-methods approach, the evaluation included a quantitative survey, in-depth interviews and focus group discussions with health workers and traditional birth attendants. Mean values of the variables of interest were compared across sub-populations, and correlation analyses were performed between changes in organizational skills and changes in relationships.

**Results:**

The results demonstrate that the Quality Circles intervention had positive effects on organizational skills and relationships. Furthermore, improvements in all organizational skill variables – problem-solving, strategizing and negotiation skills – were strongly associated with a change in the overall relationship variable.

**Conclusions:**

The Quality Circles approach has the potential to support health workers to improve their organizational skills and relationships, which in turn can contribute to improving the interpersonal dimensions of the quality of care in low-resource contexts. This method brings together peers in a structured process for constructive group work and individual skill development, which are important in low-resource contexts where active participation and resourcefulness of health workers can also contribute to better health service delivery.

## Background

One of the main challenges in delivering essential and life-saving interventions for improved maternal, newborn and child health (MNCH) outcomes is the shortage of a well-trained, supervised, and motivated health workforce [1-3]. Many low-income countries do not plan for and support nurses, midwives and community health workers to deliver MNCH interventions that improve health outcomes and save lives [4]. Sierra Leone has among the highest child and maternal mortality rates in the world despite progress in recent years [5,6]. Prior to the Ebola outbreak in 2014, Sierra Leone ranked globally among the 20 countries with the most serious health worker shortages, well below the minimum threshold set by the WHO of 2.28 health care professionals per 1,000 population [7]. The national per capita density for doctors, nurses and midwives has fallen since 2004 and is below one-sixth of that recommended by the World Health Organization [8]. The shortage of doctors and registered nurses is especially acute in rural areas, where 63 percent of the population resides [5].

Challenges related to the health workforce identified by the Government of Sierra Leone include low salaries, inadequate career advancement options and flaws in the management of the recruitment process [8]. In terms of health worker retention, challenges also include: instances of non-implementation of incentive policies for health workers and facilities to benefit when certain targets are met, problems with personnel management such as unclear decision-making for new postings and poor relationships, and inadequate systems to enhance motivation and improve working conditions [9].

Investments in public health in Sierra Leone have been predominately to increase demand for services, with fewer initiatives targeting supply side factors that influence health workers’ work environment. Driven by high mortality rates, evidence of financial barriers to care, and other factors, the Free Health Care Initiative (FHCI) was enacted in Sierra Leone in 2010 to provide free care for pregnant women, lactating mothers and children under five years of age [10,11]. A few years following the availability of essential care packages for children and women without cost throughout the country, improved outcomes were documented for some areas of maternal and child health [6,12]. In addition, in an effort to support supply side factors, the Government of Sierra Leone introduced a performance-based financing (PBF) scheme in 2011 to incentivize health providers to manage higher caseloads and increase their productivity, ultimately aiming to improve service utilization and coverage [11]. While the PBF in Sierra Leone provides disbursements to individual health workers and health facilities that meet performance targets, these are inadequate to address many issues relating to supply, demand and quality of services in many health facilities. Moreover, poor performing facilities that do not qualify for PBF incentives do not receive needed inputs.

Due to a weak public health system in Sierra Leone, which includes a scarcity of health workers and strong traditional beliefs, Traditional Birth Attendants (TBAs) continue to play influential roles in maternal and child health care in communities and sometimes in unofficial ways alongside health workers in facilities. Although rates of skilled, facility-based births have increased in Sierra Leone since the inception of FHCI, more than one-third of births are still attended by TBAs outside of health facilities [6]. In addition, many children still do not receive recommended treatment for common childhood illnesses and some are given traditional treatment instead of seeking clinical care [12,13].

The role of TBAs appears to be changing due to the launch of the FHCI in Sierra Leone with some TBAs “attached” to, or registered with, health facilities. This practice is not officially recognized at the national level, despite occurring across the country. “Unattached” TBAs continue to work independently with no relationship with health facilities [14]. Traditional Birth Attendants have been poorly integrated into this new approach to service delivery. They have no formal roles in relevant policies despite their large numbers in Sierra Leone, the fact that some of them are working with health facilities, and despite evidence from low-income countries that involving TBAs can improve rates of skilled birth attendance [15].

In addition to the lack of appropriate skills and low competencies of health care providers, motivational and relational factors of health workers are understood to be common bottlenecks to quality of care in health service delivery [16]. Lack of motivation of health providers working in low resource contexts can be challenging to address given that motivation has diverse causes and influencers; one study in Tanzania indicated that the main factors contributing to demotivation among health care workers working at primary health care facilities were heavy workload paired with staff shortages, a lack of inter-professional exchange and a lack of positive supervision [17].

Quality Circles, a method first developed in industrial work settings, has been expanded to the health sector and implemented in many countries in an effort to make rapid improvements in health care [18,19]. Quality Circles bring together groups of practitioners from different health care organizations or departments to work in a structured way with the goal of improving one aspect of the quality of their service identified and agreed upon by the group. In resource-limited settings, this and other similar methods of collaborative improvement approaches have generated evidence of producing sustained gains in compliance with standards and outcomes [20,21]. The project discussed in this paper adapted the Quality Circles approach and aimed to improve the motivation and performance of health workers and TBAs and to improve clients’ perception of quality of care in Kailahun District of Sierra Leone.

There are three main objectives of this paper. The first is to examine the effect of the intervention on organizational skills and relationships among co-workers and as well as between health workers and TBAs. The second is to examine whether changes in organizational skills are associated with changes in the relationships among and between formal and informal health providers and between health providers and clients. The third is to further understand these changes through the perspectives of health workers, TBAs and clients.

## Methods

Data used in the study are from an evaluation of this quality improvement intervention, which used a mixed-method approach to investigate the extent of change across the project objectives and vis-à-vis locations in a comparison district. Quantitative and qualitative data collection for the evaluation took place between May and July 2013 by trained field workers in Kaihalun and Tonkolili districts, following a pre-test of the tools.

### Study setting and target groups

The intervention was implemented in Kailahun District in the eastern province of Sierra Leone from August 2011 to June 2013. As of 2010, the district had a total population of 421,287 [22]. Out of a total of 159 health workers listed as working in 77 primary care health facilities, called Peripheral Health Units (PHUs), 155 health workers representing all PHUs in the district were enrolled in the project. Several cadres of health workers were included: community health officers, maternal and child health aides, state-registered nurses, vaccinators and state-enrolled community health nurses. A total of 297 TBAs were randomly selected for the project from the lists of TBAs maintained by PHUs.

### Description of the intervention

Health workers and TBAs were engaged in three tiers of “circles” to identify issues and areas for improvement in the health system, and then to implement action plans or “change ideas” to address the identified concerns. The first tier, “peer circles”, involved groups of eight to ten health workers or TBAs who gathered at monthly meetings, followed by “cross-learning circles”, which brought representatives from circles of health workers and TBAs together every other month to exchange ideas on key issues across circles. Finally, “district sharing circles” brought representatives together from selected circles across the district with the District Health Management Team (DHMT) of Kailahun to present their group’s idea, and thereafter, update participants on progress and advocate on issues they could not resolve on their own. The peer circles were the fundamental unit of the model during which external facilitators supported the participants to develop problem-solving, strategizing and negotiation skills to identify and resolve issues in the local health system.

**Figure 1:**
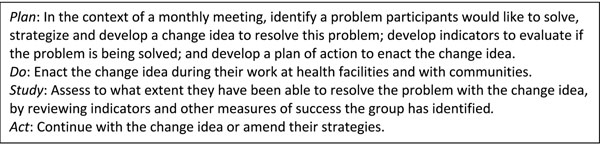
Quality circles method cycle for peer groups

The monthly meetings focused on a collaborative problem-solving methodology developed by the Institute for Healthcare Improvement, which followed a Plan-Do-Study-Act (PDSA) cycle, described in Figure 1. Each peer circle carried out this cycle at least once related to their work but was not restricted further, i.e., the idea did not have to exclusively be based on MNCH issues or facility-based care. If the circle decided their strategy was not suitable, they were encouraged to *adapt* or *abandon* the idea and move on to a different identified problem.

### Data

The quantitative component included a health worker survey and a TBA survey, designed based on the topics covered in the modules that the facilitators used to implement the project. This was also a self-reported unidimensional instrument that aimed to understand the changes in practice of relevant knowledge and skills. These surveys were administered verbally by data collectors speaking the local language (Krio) in the intervention and the control sites. Practice of organizational skills, relationships, and job satisfaction and motivation were rated by respondents on a four-point Likert scale. One module of the questionnaire included a set of retrospective pre-test questions and asked respondents to rate their skills and relationships two years prior to the survey.

Respondents were randomly selected from samples of health workers and TBAs who had been engaged in the intervention, and from samples of health workers and TBAs in the comparison district with similar socio-demographic backgrounds and from a similar mix of professional cadres. A total of 97 health workers from across 67 PHUs and 92 TBAs who participated in the intervention were included in the final sample for the evaluation in Kailahun. For the comparison, 121 health workers from PHUs Tonkolili district as well as 85 TBAs were interviewed; Tonkolili district was matched as a suitable control site following an analysis of health service structure, MNCH indicators and infrastructure challenges. The interviews took place in and around the facility premises, in a location chosen to ensure privacy and comfort.

Qualitative data from in-depth interviews (IDIs) with health workers and TBAs, focus group discussions (FGDs) with clients, and discussions with health worker and TBA peer circles, were used to contextualize quantitative findings. A total of 21 semi-structured IDIs were conducted in Kailahun from a purposeful sample of health workers (n=9) and TBAs (n=12). Additionally, six FGDs in Kailahun and six FGDs in Tonkolili were undertaken with clients. Pregnant women or women with a child under six months of age were recruited for FGDs from the catchment community of randomly selected health facilities. Group discussions with peer circles from each of the implementation phases were also conducted. The selection of the circles was done purposively in consultation with project staff and key informants in the health facilities. Three discussions with PHU staff peer circles and three discussions with TBA peer circles were conducted. Qualitative data collection was conducted by a team of trained field staff.

### Variables measurement

The outcome variables of interest to this analysis were organizational skills and relationships defined and measured as follows (see Additional file 1 for the full list of variables):

• Post-test and retrospective pre-test organizational skills in the health worker survey: Measure of practice of organizational skills as captured in 14 variables within three core domains: strategizing (five variables), problem-solving (four variables), and negotiation (five variables). All variables were defined on a 4-point Likert scale ranging from “never” to “always.” Four aggregate variables from the health worker survey on organizational skills (strategizing skills, problem-solving skills, negotiation skills, and overall organizational skills) were analyzed, all constructed as averages of responses to the corresponding individual questions, with the very few missing values imputed to the overall mean.

• Post-test and retrospective pre-test relationships in the health worker survey: Measure of health workers’ self-reported relationships with supervisor, co-workers at health facility, co-workers from other health facilities nearby, TBAs working in the community, and patients who come to the facility. The five items were also measured using a 4-point Likert scale ranging from “good” to “bad.” In the analysis, the categories were re-ordered from “bad” to “good” and ensure that higher values reflected better outcomes. One aggregate variable from the health worker survey on relationships following the procedure described above, and also imputing the few responses in the category “no relationships” to the overall mean was analyzed. Three individual variables on relationships with co-workers at facility, with TBAs, and with clients who come to the facility, were also included in the analyses.

• Post-test relationships in the TBA survey: Measure of TBAs’ report on relationships with supervisor, co-workers at health facility, co-workers from other health facilities nearby, TBAs working in the community, and patients who come to the facility. The five items were measured with a 4-point Likert scale ranging from “good” to “bad.” As above, the categories were re-ordered from “bad” to “good” to ensure that higher values reflect better outcomes across all individual variables. One individual variable from the TBA survey on relationships of TBAs with health workers at the facility was analyzed.

The Cronbach’s Alpha test for internal consistency and reliability was performed on some of these variables, and the scale reliability coefficient was 0.7204, indicating a reasonably good level of reliability, which suggested that the questions were understood and responded to correctly. In constructing the aggregate variables, the very few missing values or non-applicable values were imputed to the individual mean (i.e. 2.5). Five variables (out of the nearly 20 listed in Additional File 1) were concerned, and each had fewer than three missing or non-applicable cases.

Qualitative data were translated and transcribed verbatim into Microsoft Word documents and quality checked. Guided by the outcomes stated in the results framework, qualitative data were manually coded by the evaluation team, as described below. Qualitative data were then imported into Dedoose, an online qualitative data software for analysis.

### Analysis

The descriptive analysis compared mean values of the organizational skills and relationships variables across the three sub-populations, namely the retrospective pre-test (for intervention respondents only), the post-test and the control area. A chi-squared test was used to assess the statistical significance of the differences. Key to the analysis was the investigation of whether improved organizational skills led to improved relationships. We tested this hypothesis using correlation analyses between changes in organizational skills from the pre-test to the post-test, and changes in relationships from the pre-test to the post-test. As is common in studies on human social behaviors, the following cut-off points were used to characterize the strength of the associations: If |R| ≤ 0.30, there is a weak linear relationship; if |R| ∈[0.30, 0.65], there is a moderate linear relationship; and if |R| ≤ 0.65, there is a strong linear relationship, where |R| denotes the absolute value of the correlation coefficient. SPSS Statistics 21 was used for the analysis.

Analysis of qualitative data was carried out by a team of two researchers. Data from each set of respondents were excerpted based on the key outcomes of interest and the emerging categories and themes to understand dominant issues and patterns. In additional rounds of coding, open-ended codes were added to the coding structure.

### Ethical considerations

The Government of Sierra Leone Ethics and Scientific Review Committee granted ethical approval for the evaluation of the project. All respondents verbally gave consent to the interview and the audio recording of their responses. The consent forms also guaranteed the anonymity and confidentiality of the responses.

## Results

### Key characteristics of health workers

In general, the demographics of PHU respondents from both districts were similar (Table 1). There were fewer female respondents in Kailahun (51.5%) than in Tonkolili (63.7%). There was little difference in age distribution and mean age, though respondents in Kailahun were slightly older. Differences in marital status were minimal, but a slightly higher proportion of single respondents in Tonkolili, and correspondingly, a higher proportion of married respondents in Kailahun. Most health workers in both districts were educated to secondary level.

**Table 1:** PHU health worker respondent characteristics

	Intervention site - Kailahun % (n)	Control site - Tonkolili % (n)
Sex		
Female	51.5 (50)	63.7 (100)
Age		
Mean age	40.4 (8.2 sd)	38.1 (8.4 sd)
<25 years	1.0 (1)	3.8 (6)
25 – 34 years	19.6 (19)	29.3 (46)
35 – 44 years	47.4 (46)	43.3 (68)
≥45 years	26.8 (26)	23.6 (37)
Missing	5.2 (5)	0.0 (0)
Education		
Primary	2.1 (2)	1.3 (2)
Secondary	78.3 (76)	79.0 (124)
Tertiary and other	16.5 (16)	19.7 (31)
Missing	3.1 (3)	0.0 (0)
Marital status		
Married	81.4 (79)	70.1 (110)
Single	9.3 (9)	19.7 (31)
Other	9.3 (9)	10.2 (16)
**N**	**97**	**157**

### Descriptive analysis

Table 2 compares mean values of organizational skills and relationships variables across the pre-test, the post-test and the control site. In the post-test period, mean values of all four organizational skills variables appeared statistically higher in the intervention district than in the control site (p<0.001), with the greatest difference recorded for strategizing skills (difference of 0.8 points). The strategizing dimension included variables such as the ability to identify areas where the facility is doing well, where the facility has problems, what is causing the problem, what can help solve the problem, and why it can be difficult for the facility to solve the problem. The pre-test to post-test differences in strategizing skills were also positive and statistically significant, reaching 1.06 points on organizational skills (p=0.000) and 1.01 on problem-solving (p=0.005).

**Table 2:** Health worker organizational skills and relationships

	Average levels			Post-Test Pre-Test difference		Post-Test Control difference	
			
Variable	Pre-Test (Kailahun)	Post-Test (Kailahun)	Control (Tonkolili)				
	(1)	(2)	(3)	(2)-(1)	P-value	(2)-(3)	P-value
Health Workers' Organizational Skills							
Strategizing skills	2.65	3.72	2.91	1.064	0.000	0.808	0.000
Problem solving skills	2.94	3.95	3.19	1.010	0.005	0.765	0.002
Negotiation skills	2.73	3.59	3.09	0.860	0.000	0.505	0.000
Overall organizational skills	2.76	3.74	3.05	0.976	0.000	0.687	0.000
Health Worker Relationships							
With co-workers at facility	2.86	3.62	3.44	0.763	0.000	0.179	0.016
With patients	2.82	3.53	3.49	0.706	0.000	0.035	0.667
With TBAs	2.42	3.49	3.24	1.077	0.000	0.259	0.007
Overall relationships**^1^**	2.73	3.55	3.35	0.823	0.000	0.196	0.002
**N**	**97**	**97**	**157**				
TBA Relationships							
With health workers at facility	NA	3.58	3.05	NA	NA	0.534	0.000
**N**	**NA**	**92**	**85**				

**^1^**Also include relationships with supervisors and with co-workers from other facilities.

Table 2 also shows that the aggregate relationships variable from health workers’ perspectives was only slightly higher in the intervention (Kailahun) district post-test, compared with the control area (difference of 0.196) although this difference was statistically significant (p=0.002). Noticeably, there was no difference in post-test across the two areas with regard to relationships of health workers with patients, and only a small, positive, statistically significant difference in the relationships with co-workers (p=0.016). The largest difference between the intervention and the control communities was recorded on the relationship of health workers with TBAs (difference of 0.259 points, p=0.007). Within the intervention district, the changes from pre-test to post-test in health workers’ relationships were all positive and statistically significant (p=0.000), with the largest difference noted on the relationships with TBAs as in the intervention-control comparison described above.

Finally, according to the TBA survey, the relationships with health workers were reported to be better in Kailahun, compared to Tonkolili (p=0.000), corroborating the results from health workers on their relationships with TBAs. As indicated earlier, the TBA survey did not include a retrospective assessment of relationships.

### Correlation analysis

Table 3 presents the correlation coefficients between changes over time in the organizational skills variables and changes over time in the three relationships variables among health workers in the intervention district. All coefficients are positive indicating, where statistically significant, that increased skills from pre-test to the post-test were linearly associated with improved relationships from pre-test to the post-test.

**Table 3:** Correlation coefficients between changes in skills and changes in relationships of health workers

	Change in Relationships			
	
	With Co-workers	With Patients	With TBAs	Overall
Change in Strategizing skills	.164	.274^**^	.097	.280^**^
	97	97	97	97

Change in Problem-solving skills	.233^*^	0.122	.096	.316^*^
	97	97	97	97

Change in Negotiation skills	.367^**^	.328^**^	.185	.417^**^
	97	97	97	97

Change in Overall organizational skills	.303^**^	.228^*^	.139	.405^**^
	97	97	97	97

^**^Correlation is significant at the 0.01 level (p<0.01)

^*^Correlation is significant at the 0.05 level (p<0.05)

As the last row of Table 3 shows, the correlation between change in overall organizational skills and change in relationships with co-workers is strong (+0.303, p<0.01), while that between change in overall organizational skills and change in relationships with patients is weaker (+0.228, p<0.05). Improvement in the overall organizational skills variable is strongly associated with improvement in the overall relationships variable (+0.405, p<0.01). Noticeably, improvements in all organizational skills variables are strongly associated with change in the overall relationship variable (see last column of Table 3).

While Table 2 shows a significant improvement over time in the relationships of health workers with TBAs from ratings ranging from 2.42 to 3.29 (p=0.000), the correlation analysis indicates that this improvement is not associated with reported improvements in organizational skills from an average of 2.76 to 3.74 (p=0.000). Importantly, the improvement in the relationships with TBAs is not associated with changes in any of the three organizational skills categories (Table 3). Among the three components of organizational techniques, changes in negotiation skills and problem-solving skills have strong correlations with changes in relationships with co-workers (+0.367 and +0.233, respectively), while improvements in negotiation skills and in strategizing skills have strong correlations with relationships with patients (+0.328 and +0.274, respectively).

### Qualitative insights into organizational skills and relationships

While Quality Circles activities were directed towards developing new skills and improving existing problem-solving skills among PHU staff, being a part of the group process was in itself expected to strengthen relationships. Through IDIs and FGDs, health workers in the intervention district reported that project activities helped them to analyze their ability to effect change and to make that change happen, through improved organizational skills and improved relationships.

In most interviews, respondents from the intervention area referred to the Quality Circles tools for organizational skills including problem-solving, in particular using concepts and tools introduced by the project, to delve into the different factors that prevented effective health service delivery and to analyze root causes of a particular problem. During interviews, health workers were able to easily describe some of the problems identified and strategies developed to overcome them through the Quality Circles methodologies.

Health workers indicated that the choice of priority problems to be addressed involved considerable discussion within the group and required consensus building to guarantee the buy-in of every participant and increase the likelihood that the change ideas could be successfully achieved. Health workers also reported that implementation of the solutions involved consultations and negotiations with other stakeholders, most importantly with TBAs, as many change ideas sought to clarify their role and participation in the local health system. Communities and clients were also reported to have been included in the implementation of the strategies so that change ideas received approval and support of key influencers, such as supervisors or community leaders.

Staff shortages were frequently reported as a barrier to efficient service delivery and many circles identified greater involvement of local resources, namely TBAs, to support day-to-day operations and tasks. Guided by the project processes, health workers were able to identify tasks that could be shifted to TBAs, who were considered appropriate to provide support with both non-technical activities such as facility cleaning and maintenance, as well as more clinical tasks such as growth monitoring of infants, though one health worker made it clear that adequate training would need to be provided:

*“Because we have insufficient staff, I brought up a suggestion that we should train TBAs to be assisting with the growth monitoring, that is weighing the babies, and one of the staff members stood against the point that some of the TBAs will not understand how to read the scale and how to weigh. But the in-charge came in and we look[ed] amongst the TBAs for someone who [could] read or write and we train[ed] her up, and now she is working with me.”* (Health worker, Ngehun, Luawa)

Among various work-related relationships PHU staff have, their relationships with TBAs emerged as an area where important changes and improvements were reported. Relationships with other PHU co-workers were generally reported to be positive, but any changes in this relationship as a result of the project were more muted as illustrated by one health worker:

*“Well, we are all individuals, we are all human being[s], no matter what you do, one way or the other, you might offend another person. But not withstanding that, we can still count out our indifferences.”* (Health worker, Daru, Jawie)

However, others demonstrated support for others in their responses:

*“I have a very good relationship with them [TBAs] because if I have a problem in my [health] center, I will call other health workers in other centers and they will come to help me and also if any one of them has a problem, as long as I know about the problem, I will go there to give my help. So that is the way we are working.”* (Health worker, Fola, Njaluhuahun)

Across all IDIs and FGDs, health worker respondents refer to a “revival” of relationships with TBAs and the support they receive from them, linked to the categories of organizational skills and relationships. Reports suggest that PHU staff have always had a working relationship with some TBAs who used to assist with deliveries at the facilities. The introduction of the Free Health Care Initiative and the related opposition to TBA-led home deliveries was reported to have recently pushed some TBAs to dissociate themselves from the facilities and continue their childbirth services privately, with no interaction with the formal health system, while other TBAs were reported to have continued working with health facilities in different lower-skilled capacities.

## Discussion

Overall, the results demonstrate that the Quality Circles intervention had a positive effect on health workers’ organizational skills and relationships. There was an increase in self-rating of organizational skills among health worker respondents who participated in the intervention compared with retrospective responses and compared with health workers from the control district. These findings indicate that the Quality Circles methodology can produce the desired outcome of self-perceived improvements in organizational skills of health workers, which include strategizing, problem-solving and negotiation at work. Of these skills, strategizing, which contains variables linked to identifying problems, causes and solutions, and relates directly to the group work technique of Quality Circles to plan, implement, review and revise an idea, saw the largest improvement. This finding is supported by existing evidence on the effectiveness of peer-based quality improvement techniques in low-resource contexts [20], as well as by identified linkages of active participation on health workers’ self-efficacy, the “can-do” component of health worker motivation [23].

Improvements occurred for relationships in the intervention district across all variables between pre- and post-test and vis-à-vis the comparison, with the largest difference reported for relationships with TBAs. This finding was supported by the qualitative portion of the study, which found improvements in relationships especially between health workers and between health workers and TBAs. Quality Circles as a methodology enhances factors related to relationships with peers and supervisors both through participation of team members in these groups and through the acquisition of skills to negotiate various situations. Corroborating the improvement in relationships reported by health workers in the intervention area, TBAs indicated that their relationships with health workers are better in the intervention site compared to the pre-test and the control. This illustrates the two-way perceptions of improvements between formal and informal health workers that changed after the introduction of Quality Circles. While the format of the intervention involved interactions among health workers and TBAs separately, the cross-learning circles allowed interaction between representatives of the groups. Moreover, many of the ideas of both formal and informal health workers conceptualized, implemented and reviewed during the project related to ways to involve TBAs in some of the functions of the health facility or in community health awareness or outreach.

The correlation analyses were important to understand where associations existed between the variables; these analyses showed that the change in overall organizational skills and the change in overall relationships were strong, particularly with the variable measuring relationship with co-workers. This is likely due to the nature of the intervention and how it enabled interactions related directly to organizational skills between and relationships among co-workers. Also, changes in negotiation skills and in problem-solving skills showed strong correlations with change in relationships with co-workers, which may indicate that better negotiation and problem-solving skills can reinforce positive relationships among co-workers.

Given that interactions with individual patients are not usually as frequent as routine interactions between a health worker and her/his co-workers, especially with the addition of co-worker interaction through the Quality Circles groups, it is not surprising that there is a weaker correlation between overall organizational skills and relationships with patients. It could have been expected there would be some spillover effect from improved organizational skills of health workers to their relationships with patients; however, data from this study indicate this was not the case. Still, changes in the variables of negotiation skills and strategizing skills had strong correlations with health workers’ perceived relationships with patients.

While one finding revealed improvements in relationships between health workers and TBAs, these improvements were not associated with changes in any of the three organizational skills variables of health workers. This is an interesting finding that suggests that the relationships changed for other reasons. As some circles identified utilizing TBAs for different functions at the health facility, it is possible that the change in relationships was not related to the health workers’ individual sense of improved organizational skills, but rather to health workers’ acknowledgement that TBAs’ experience as well as their linkage with their communities are an important part of the extended health system and that they should be integrated in some way. Cross-learning circles – periodic groups of representatives of the 63 groups of health workers and TBAs – were another way in which these relationships could have improved.

The findings of this study add two key contributions to the literature on collaborative improvement approaches generally and to the Quality Circles technique specifically. First, the approach appears to result in self-perceived improvements in skills of individual health workers important to dimensions of the provision of quality of care. Assessment or measurement of the quality of care has been studied for decades [24,25], including more recently for obstetric care in low-resources contexts [26,27]. This body of research systematically invokes the importance of human resource dimensions (e.g., staffing, technical performance, etc.) in the quality of care, including interpersonal aspects of care. These studies call for quality improvement approaches that involve interpersonal skill development, often focused on communication alone. Findings from this study suggest that the process of engaging health workers in peer groups can improve their relationships and organizational skills, which may also contribute to improvements in communication, a closely related skill.

A second contribution relates to the inclusion of informal health workers, which is a unique feature of the implementation of this approach not previously studied. In a country such as Sierra Leone, which has a low ratio of qualified health personnel to population and a customarily heavy reliance on traditional care, this method acknowledges TBAs as an inevitable part of the health system and with potential contributions to its improvement. Indeed, evidence indicates that TBAs have potential to be involved in delivering some MNCH interventions [28,29] including counseling [30], and to perform a liaison function with other health workers [31]. While observations on the roles and potential roles of TBAs in Sierra Leone is outside the scope of this paper, this study does indicate that a peer group model is a structured format to constructively find ways to involve informal health workers, especially where resources are limited, as well as a way to build relationships and other skills across different groups within a local health system.

Overall within low-resource settings, engaging groups of frontline health providers in quality improvement approaches is a relatively inexpensive methodology to begin to address quality of care issues. Project Fives Alive, a collaborative learning model in Ghana which aimed to generate quality improvements in MNCH services through facilitating the involvement of frontline health workers in quality improvement teams, scaled to national level following evidence of improvements in health outcomes [32]. Even in low-resource contexts characterized by insecurity, such as Afghanistan, measurable improvements in patient care at the frontlines of service delivery can be made while systematically building capacity through a peer group quality improvement approach [21]. Though this study did not measure changes in health outcomes, evidence produced suggests this approach is a viable strategy to improve health providers’ organizational skills and relationships and could be effective in addressing skills of health workers alongside other strategies for health systems strengthening. Sustainability of quality improvement projects such as this one can be through formal incorporation into national health systems; however, faced with many challenges, limited resources and a growing number of quality improvement approaches and techniques to choose from, adoption may not figure among health sector priorities.

### Limitations

Limitations in this paper include potential biases associated with retrospective questioning, as well as with surveys of self-assessment of skills and attributes. Although nearly all health workers in the district were recruited for the survey, the district has a relatively small health workforce, thus affecting sample sizes.

## Conclusion

The Quality Circles approach has the potential to support health workers in organizational skills and relationships, which contribute to improvement of interpersonal dimensions of quality of care. This approach can be particularly useful in low-resource contexts where active participation and resourcefulness of health workers can contribute to better health service delivery. While some ambiguity remains on linkages between informal providers and the formal health system at the policy level, TBAs continue to be a strong force at the community level in Sierra Leone and they have the potential to play a variety of roles in the health sector, from community awareness, to linking with facility-based care, to engaging in non-clinical tasks within the facility. This approach to quality improvement could also be considered in recovery from a humanitarian crisis such as the Ebola virus outbreak, which has directly and intensely affected health workers; it could be used to bring together peers in a structured process for constructive group work and individual skill development.

## Abbreviations

FHCI: Free Health Care Initiative in Sierra Leone; MNCH: maternal, newborn and child health; PBF: performance based financing; PDSA: *Plan-Do-Study-Act* cycle, a collaborative problem-solving methodology developed by the Institute for Healthcare Improvement; PHU: Peripheral Health Unit, primary care health facilities in Sierra Leone; TBAs: Traditional Birth Attendants

## Competing interests

The authors declare that they have no competing interests.

## Authors’ contributions

AHS contributed to development of data collection methods and tools, drafted and revised the manuscript. JCF oversaw development of data collection methods and tools and analyzed the quantitative data. KW contributed to qualitative data analysis and literature review. LV contributed to the literature review. All authors reviewed the manuscript in detail on several occasions and approved the final product.

## Supplementary Material

Additional file 1 supplementary material. Click here for file

## Declarations

The publication costs for this article were funded by the *Innovations for Maternal*, *Newborn & Child Health*, an initiative funded by Bill & Melinda Gates Foundation.

This article has been published as part of *BMC Health Services Research* Volume 15 Supplement 1, 2015: Improved access to maternal, newborn and child health services: strengthening human resources for health. The full contents of the supplement are available online at http://www.biomedcentral.com/bmchealthservres/supplements/15/S1.
