# Supplementary material for: Peer-driven quality improvement among health workers and traditional birth attendants in Sierra Leone: linkages between providers’ organizational skills and relationships
Source: BMC Health Serv Res. 2015 Jun 8;15(Suppl 1):S4. doi: 10.1186/1472-6963-15-S1-S4 (PMC4464036; doi:10.1186/1472-6963-15-S1-S4)
Supplement: Additional file 1 — supplementary material. [file 1472-6963-15-S1-S4-S1.docx]

**Annex 1. Selected sections of the survey questionnaires**

**1. Organizational skills – Health worker survey**

| **…. would you say** | Never | Not often | Often | Always |
| --- | --- | --- | --- | --- |
|  | **1** | **2** | **3** | **4** |
| ***Strategizing*** | | | | |
| 1. You are able to tell what the facility is good at doing |  |  |  |  |
| 1. You are able to tell what the problems are at the facility |  |  |  |  |
| 1. You know what is causing the problem |  |  |  |  |
| 1. You are able to think about what can help solve the problem |  |  |  |  |
| 1. You are able to understand why it can be difficult for the facility to solve the problem |  |  |  |  |
| ***Problem solving*** | | | | |
| 1. You are able to talk about the problem with co-workers |  |  |  |  |
| 1. You are able to confidently tell what you feel should be the solution |  |  |  |  |
| 1. You are able to respect someone else’s viewpoint |  |  |  |  |
| 1. You know who to talk to if you need more information about solving the problem |  |  |  |  |
| ***Process negotiation*** | | | | |
| 1. You are able to talk about the problem with supervisors |  |  |  |  |
| 1. You are able to directly or through your co-workers tell the DHMT about the facility’s problem |  |  |  |  |
| 1. You are able to agree to what others say |  |  |  |  |
| 1. You are able to adjust your ideas to allow changes to happen at the facility |  |  |  |  |
| 1. You are able to talk to the community about the problems that the facility faces |  |  |  |  |

**2. Relationships – Health worker and TBA surveys**

| **… what would you say about …** | Very good | Good | Moderate | Bad | No relation-ship |
| --- | --- | --- | --- | --- | --- |
|  | **1** | **2** | **3** | **4** | **5** |
| 1. Your relationship with your supervisor |  |  |  |  |  |
| 1. Your relationships with your co-workers at your health facility |  |  |  |  |  |
| 1. Your relationship with co-workers from other health facilities nearby |  |  |  |  |  |
| 1. Your relationship with TBAs working in your community |  |  |  |  |  |
| 1. Your relationship with patients who come to the facility |  |  |  |  |  |
